# Awareness of Stroke Risk Factors, Warning Signs, and Preventive Behaviour Among Diabetic Patients in Al-Ahsa, Saudi Arabia

**DOI:** 10.7759/cureus.35337

**Published:** 2023-02-22

**Authors:** Ahmed Elshebiny, Mohammed Almuhanna, Mohammed AlRamadan, Mohammed Aldawood, Zuhair Aljomeah

**Affiliations:** 1 Internal Medicine, Diabetes and Endocinology, Faculty of Medicine, Menoufia University, Shebin El Kom, EGY; 2 Internal Medicine, Diabetes and Endocrinology, College of Medicine, King Faisal University, Al Hofuf, SAU; 3 Neurology, College of Medicine, King Faisal University, Al Hofuf, SAU; 4 Internal Medicine, College of Medicine, King Faisal University, Al Hofuf, SAU; 5 General Surgery, College of Medicine, King Faisal University, Al Hofuf, SAU

**Keywords:** stroke risk, ischemic and hemorrhagic stroke, macrovascular complication, risk factors, symptoms, warning signs, diabetes, stroke

## Abstract

Objectives

This study aims to measure the level of awareness about stroke symptoms, risk factors, and preventive health practices that could be taken to reduce the risk of stroke among diabetic patients in Al-Ahsa, Saudi Arabia.

Methods

A cross-sectional study was conducted in Al-Ahsa, Saudi Arabia in 2020. The sample included a total of 202 male and female Saudi adults aged 18-65 years, with either type 1 or type 2 diabetes mellitus, and living in Al Ahsa, Saudi Arabia. The information was collected randomly through an online questionnaire distributed among patients after getting their contact information from relevant governmental and private diabetes clinics and after signing the informed consent.

For awareness and knowledge items, each correct answer was scored one point and the total summation of the discrete scores of the different items was calculated. A diabetic patient with a score less than 60% of the total score was considered to have poor awareness while a score of 60% or more of the total score was considered a good level of awareness.

Results

A total of 87 (43.1%) participants had an overall good awareness level, while 115 (56.9%) had poor awareness levels. Around 40.6% of the study patients had heard about stroke, 61.9% knew that stroke affects the brain, and 24.3% reported that stroke is higher among males. As for factors associated with stroke, the most reported was high blood pressure (71.8%), followed by diabetes mellitus (69.3%). Exactly 65.8% of participants knew about the mechanism of ischemic stroke and 42.6% reported hemorrhagic stroke. A high percentage of patients (73.1%) realize that they could reduce their risk of stroke.

Conclusion

The findings of the current study showed that less than half (43.1%) of the Saudi patients with DM had a good awareness level regarding stroke and its related risk factors and warning signs. Older patients (aged 50-65 years) with high social levels (high education and income) and those with a family history of stroke had significantly higher awareness levels. Hypertension, DM, and smoking are the highest reported known risk factors of stroke, and speech disorders are the highest known stroke presentation to the respondents.

## Introduction

Diabetes mellitus (DM) is a metabolic disorder characterized by either insulin resistance or the inability of the body to produce a proper amount of insulin or both, resulting in hyperglycemia [1,2]. The prevalence of DM is estimated to be 34% in the Saudi population, while the global prevalence of DM is about 9.3%. The Kingdom of Saudi Arabia has the seventh highest DM prevalence [3-5].

DM is considered an independent risk factor for stroke due to the fact that DM causes vascular aging, which leads to microvascular and macrovascular complications. Stroke is a neurological condition, which occurs abruptly due to a pathology in the vessels of the brain or in the vessels from or to the brain. DM is a chronic disease that does not only need medical attention but also requires self-care and a patient's knowledge about the potential secondary illnesses that might be caused by it [6-11].

DM is considered a major modifiable risk factor for stroke. Moreover, DM is highly associated with pathophysiological changes including vascular endothelial dysfunction, arterial stiffness, thickening of the capillary basal membrane, and systemic inflammation. Stroke is one of the highlighted macrovascular complications of DM and it is more likely to be associated with type-2 DM [6-11]. Furthermore, diabetic patients have twice the risk of developing both ischemic and hemorrhagic stroke compared with those who are non-diabetic, which makes DM accountable for almost one-quarter of all stroke cases [6-11]. In addition, diabetic patients who developed stroke have less favorable outcomes [9].

Worldwide, stroke and other cerebrovascular accidents are major health issues with a huge burden at the individual, family, and social levels [8]. Also, the financial burden is a significant issue with an estimated direct medical cost of $273-$818 billion between 2010 and 2030 in the United States alone [8]. Stroke is one of the most prevalent diseases in Saudi Arabia with a prevalence rate of 43.8 per 100,000 in Riyadh and 40 per 100,000 in the Eastern province. In Saudi Arabia, stroke is the third leading cause of death after ischemic heart disease and road injuries [12-13].

A previous study assessed the knowledge of stroke risk factors and warning symptoms among the Saudi general population and found that 63.8% of the participants had a poor knowledge level [14]. Another study conducted among stroke patients at King Abdulaziz Medical City, Riyadh, Saudi Arabia, has shown that more than 50% of the patients were unaware that they were having a stroke. Most of them sought medical care late because of failing to notice signs and symptoms [15].

Therefore, this study aimed to measure awareness levels about stroke symptoms, risk factors, and preventive health practices among patients with diabetes. Subsequently, this will lead to a reduction in the risk of stroke among these patients. The results of the study would be of help to the local health authorities in planning effective educational programs to increase the awareness of these patients. Eventually, this may reduce the burdens and costs caused by stroke.

## Materials and methods

A cross-sectional questionnaire-based study was conducted in Al-Ahsa, Saudi Arabia, from January 1, 2022, to December 31, 2022. The population included both male and female diabetic Saudi adults, 18-65 years of age, living in Al Ahsa, Saudi Arabia. All types of DM were included. The study was approved by the Research Committee of King Fahad Hospital, Hofuf, AI Ahsa, Kingdom of Saudi Arabia (Approval number: IRB KFHH (H05-HS-065) 46-33-2020).

The calculated sample size was 385 participants, determined by the Richard Geiger equation, with a margin of error determined as 5%, a confidence level of 95%, the population as 1,041,863, and 50% for response distribution. The information was collected randomly through an online questionnaire distributed among patients after getting their contact information from relevant governmental and private DM clinics. All patients signed approval to share in the study after being informed of the study details and its rationale. We obtained responses only from 202 participants.

After data were extracted, they were revised, coded, and analyzed using IBM SPSS Statistics for Windows, Version 22.0 (Released 2013; IBM Corp., Armonk, New York, United States). All statistical analysis was done using two-tailed tests. A p-value less than 0.05 was considered statistically significant. For awareness and knowledge items, each correct answer was scored one point and the total summation of the discrete scores of the different items was calculated. A diabetic patient with a score less than 60% of the total score was considered to have poor awareness while a score of 60% or more of the total score was considered good awareness.

Descriptive analysis based on frequency and percent distribution was done for all variables including patients’ demographic data, medical and family history, and smoking. Also, patients’ awareness regarding stroke, risk factors, warning signs, and prevention with preferred sources of information were tabulated and graphed. Cross tabulation was used to assess the distribution of knowledge levels according to patients' personal data, medical data, and risk factors. Relations were tested using the Pearson chi-square test and exact probability test for small frequency distributions.

## Results

A total of 202 diabetic patients completed the study questionnaire. Patients were aged 18 to 65 years with a mean age of 45.1 ± 16.6 years old. A total of 101 (50%) patients were females and 50% were males. A total of 137 (67.8%) patients were married and 73 (36.1%) were university educated while 66 (32.7%) had a secondary level of education. Considering work, 102 (50.5%) were non-healthcare workers, healthcare workers represented only 5% of the samples, and the rest are students or not working; a monthly income of less than 3000 SR was reported by 77 (38.1%) participants (Table 1).

**Table 1:** Sociodemographic data of the study participants

Socio-demographic data	No	%
Age in years		
18-24	34	16.8%
25-34	25	12.4%
35-49	57	28.2%
50-65	86	42.6%
Gender		
Male	101	50.0%
Female	101	50.0%
Marital status		
Single	65	32.2%
Married	137	67.8%
Educational level		
Below secondary	63	31.2%
Secondary	66	32.7%
University	73	36.1%
Work		
Not working / student	90	44.6%
Non-health care worker	102	50.5%
Health care worker	10	5.0%
Monthly income		
< 3000 SR	77	38.1%
3000-5000 SR	33	16.3%
5000-10000 SR	52	25.7%
> 10000 SR	40	19.8%

Exactly 95 (47%) patients had hypercholesterolemia, 88 (43.6%) were hypertensive, 46 (22.8%) had cardiovascular disease, and 32 (15.8%) were smokers for more than one year. A total of 74 (36.8%) patients had a family history of stroke (Table 2).

**Table 2:** Medical and family history of the study participants HTN: hypertension; CVD: cardiovascular disease

Medical & family history	No	%
Co-morbidities		
HTN	88	43.6%
CVD	46	22.8%
Hypercholesterolemia	95	47.0%
Smoking for more than 1 year	32	15.8%
Do you have anyone in your family who has or had a stroke		
Yes	74	36.8%
No	103	51.2%
Don’t know	24	11.9%
Have you had a stroke before?		
Yes	11	5.5%
No	182	90.5%
Don’t know	8	4.0%
If yes, how many times you had it?		
1 time	9	81.8%
> 3 times	2	18.2%

**Figure 1:**
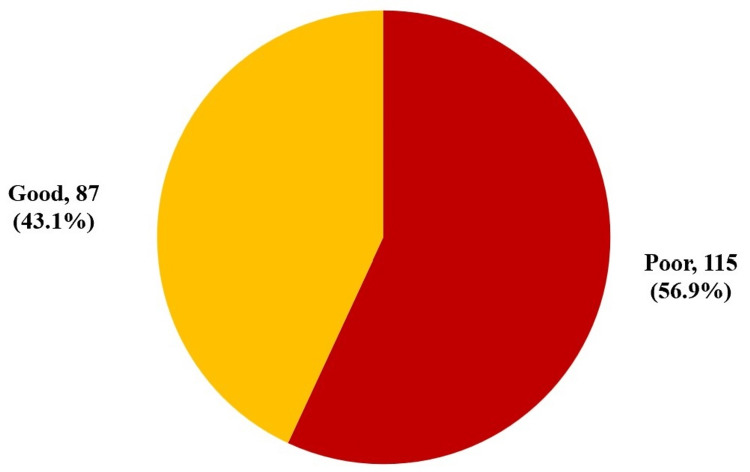
Overall awareness regarding stroke risk factors, warning signs, and preventive behaviors among the study participants

Generally, 40.6% of the study patients heard about stroke, 61.9% knew that stroke affected the brain, and 24.3% reported that stroke was higher among males. As for factors associated with stroke, the most reported was high blood pressure (71.8%), followed by DM (69.3%), cigarette smoking (64.9%), and high cholesterol level (57.4%). A total of 65.8% knew about the mechanism of ischemic stroke. Regarding clinical presentation, 70.8% mentioned speech disorders, and 69.8% knew of weakness or disability to move one-half of the body. A total of 73.1% of the patients knew that they could reduce the risk of stroke (Table 3). A total of 87 (43.1%) had an overall good awareness level while 115 (56.9%) had a poor awareness level (Figure 1).

**Table 3:** Awareness regarding stroke, risk factors, consequences, and prevention among the study participants

Domain	Items		No	%
General awareness	Know about stroke	Yes	82	40.6%
		No	120	59.4%
	Stroke is a disorder primarily affecting	Brain	125	61.9%
		Heart	18	8.9%
		Blood sugar	5	2.5%
		Don’t know	54	26.7%
	Do you think the risk of stroke is	Higher in males	49	24.3%
		Higher in females	18	8.9%
		Equal in both	37	18.3%
		Don’t know	98	48.5%
Risk factors awareness	Which of the following age groups has a higher risk of stroke?	< 30 years old	7	3.5%
		30-50 years old	50	24.8%
		> 50 years old	90	44.6%
		Don’t know	55	27.2%
	Can young people suffer a stroke?	Yes	111	55.0%
		No	19	9.4%
		Don’t know	72	35.6%
	High blood pressure	Yes	145	71.8%
		No	11	5.4%
		Don’t know	46	22.8%
	Cigarette smoking	Yes	131	64.9%
		No	30	14.9%
		Don’t know	41	20.3%
	Diabetes mellitus	Yes	140	69.3%
		No	15	7.4%
		Don’t know	47	23.3%
	High cholesterol level	Yes	116	57.4%
		No	28	13.9%
		Don’t know	58	28.7%
Mechanism of stroke awareness	Rupture of blood vessels	Yes	86	42.6%
		No	33	16.3%
		Don’t know	83	41.1%
	Blockage of blood vessels	Yes	133	65.8%
		No	13	6.4%
		Don’t know	56	27.7%
	Tension	Yes	120	59.4%
		No	24	11.9%
		Don’t know	58	28.7%
	Worrying	Yes	118	58.4%
		No	27	13.4%
		Don’t know	57	28.2%
Stroke clinical presentation awareness	Speech disorder	Yes	143	70.8%
		No	16	7.9%
		Don’t know	43	21.3%
	Weakness or disability to move one half of the body	Yes	141	69.8%
		No	18	8.9%
		Don’t know	43	21.3%
	Decreased sensation or inability to feel things	Yes	127	62.9%
		No	23	11.4%
		Don’t know	52	25.7%
	Decreased vision	Yes	130	64.4%
		No	14	6.9%
		Don’t know	58	28.7%
Prevention awareness	Person can reduce the risk of stroke?	Yes	147	73.1%
		No	5	2.5%
		Don’t know	49	24.4%

**Figure 2:**
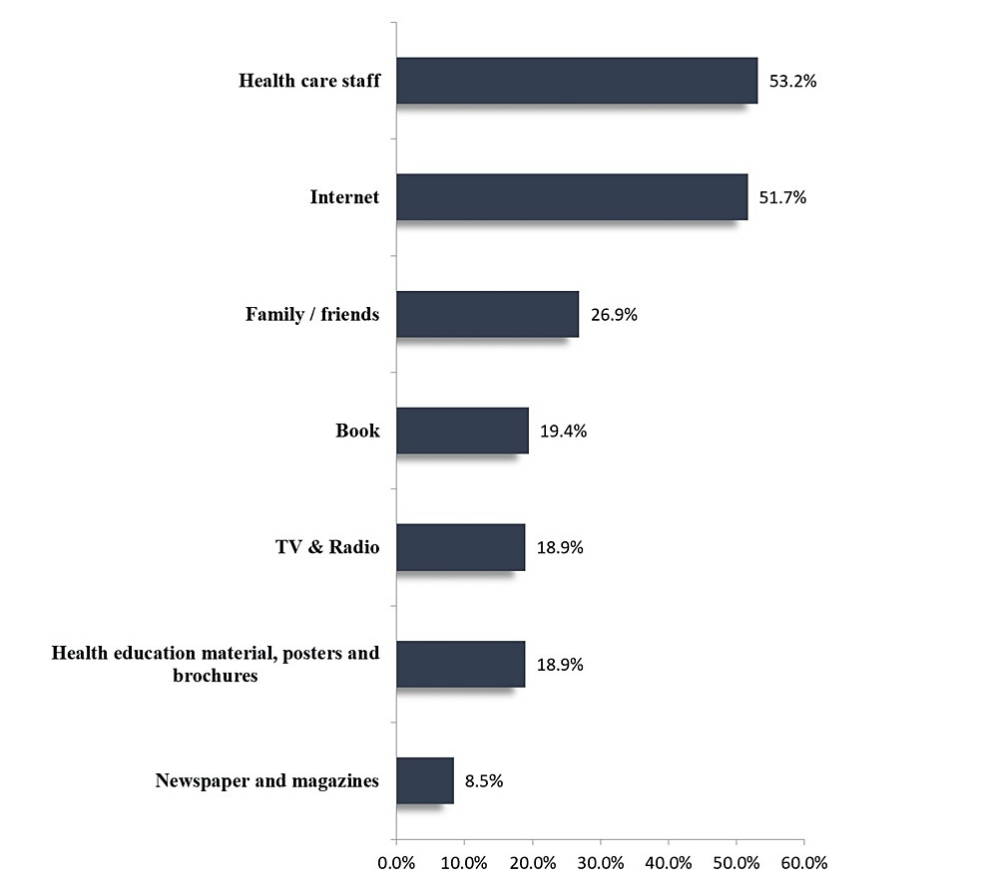
The preferred source of information regarding stroke as reported by the study patients

Exactly 52.3% of the older patients (aged 50-65 yers) had a good awareness level compared to 47.1% of the younger age group, which was was statistically significant (P-value=0.021). Also, 67.5% of patients with high income showed a good awareness level (P-value=0.004). A total of 50.5% of patients with hypercholesterolemia had a good awareness of stroke (P-value=0.044). Additionally, 51.4% of patients with a family history of stroke had a good awareness of the disease (P-value=0.012) (Table 4). The most preferred source was healthcare personnel (53.2%), followed by the internet (51.7%), family/friends (26.9%), books (19.4%), and television and radio (18.9%) (Figure 2).

**Table 4:** Factors associated with the awareness regarding stroke risk factors, warning signs, and preventive behaviors ^P^ Pearson X2 test; ^$ ^Exact probability test; * P < 0.05 (significant)

Factors		Awareness level				p-value
		Poor		Good		
		No	%	No	%	
Age in years	18-24	18	52.9%	16	47.1%	.021*
	25-34	14	56.0%	11	44.0%	
	35-49	42	73.7%	15	26.3%	
	50+	41	47.7%	45	52.3%	
Gender	Male	52	51.5%	49	48.5%	.118
	Female	63	62.4%	38	37.6%	
Marital status	Single	41	63.1%	24	36.9%	.224
	Married	74	54.0%	63	46.0%	
Educational level	Below secondary	41	65.1%	22	34.9%	.049*
	Secondary	40	60.6%	26	39.4%	
	University	34	46.6%	39	53.4%	
Work	Not working / student	54	60.0%	36	40.0%	.426
	Non-health care worker	54	52.9%	48	47.1%	
	Health care worker	7	70.0%	3	30.0%	
Monthly income	< 3000 SR	49	63.6%	28	36.4%	.004*
	3000-5000 SR	18	54.5%	15	45.5%	
	5000-10000 SR	35	67.3%	17	32.7%	
	> 10000 SR	13	32.5%	27	67.5%	
HTN	Yes	50	56.8%	38	43.2%	.977
	No	65	57.0%	49	43.0%	
CVD	Yes	24	52.2%	22	47.8%	.458
	No	91	58.3%	65	41.7%	
Hypercholestrolemia	Yes	47	49.5%	48	50.5%	.044*
	No	68	63.6%	39	36.4%	
Smoking for more than 1 year	Yes	15	46.9%	17	53.1%	.210
	No	100	58.8%	70	41.2%	
Do you have anyone in your family who has or had a stroke	Yes	36	48.6%	38	51.4%	.012*
	No	58	56.3%	45	43.7%	
	Don’t know	20	83.3%	4	16.7%	
Have you had a stroke before?	Yes	4	36.4%	7	63.6%	.228^$^
	No	104	57.1%	78	42.9%	
	Don’t know	6	75.0%	2	25.0%	

## Discussion

The current study aimed to assess diabetic patients’ awareness regarding stroke risk factors, warning signs, and prevention in Al-Ahsa, Saudi Arabia. The study showed that less than half (43.1%) of the patients had a good awareness level regarding stroke and its related risk factors and warning signs. More specifically, less than half (40.6%) of the study patients had heard about stroke. About two-thirds (61.9%) of the patients knew that stroke affects the brain, but only one-fourth of them reported that stroke is higher among males.

The literature showed that the prevalence of stroke is higher among men till the age of 80 years, after that it is higher in women. Most of the studies concluded that the case fatality rate is higher in female than in male stroke patients; there is also some evidence, albeit relatively weak, indicating a better functional outcome in men. As for factors associated with stroke, the most reported was high blood pressure, DM, cigarette smoking, and high cholesterol level [16-19].

Regarding knowing stroke different clinical presentations by the study group, 70.8% reported knowing about speech disorders, 69.8% knew about weakness or disability to move one-half of the body, 64.4% knew about decreased vision, and 62.9% reported knowing about decreased sensation or inability to feel things. A total of 73.1% of the patients knew that they could reduce the risk of stroke. The study also revealed that older patients (aged 50-65 years) with high social levels (high education and income) and those with a family history of stroke had significantly higher awareness levels. 

Arisegi et al. showed that 70.3% of diabetic patients had good knowledge of stroke, organs or parts of the body affected by stroke (89.1%), signs or symptoms of stroke (87.0%), stroke risk factors (86.6%), and stroke prevention (90.8%) [20]. Formal education was the sole predictor of good knowledge of the signs or symptoms of stroke. Studies in Nigeria [21,22], the United States [23], and Australia [24] also showed that the majority of the participants had good knowledge of stroke as a disease of the blood vessels in the brain.

Regarding warning signs and clinical presentation, a study in Ghana reported numbness or paralysis as the most common stroke warning sign known to participants [25]. While it also concurs with the findings in studies conducted in Osogbo, Nigeria [22], Benin [26], and Nigeria [27]. It differs from the findings in studies conducted in Australia [24] and Ireland [28] that reported visual problems and slurred speech, respectively, as the most common stroke signs identified. In Saudi Arabia, Alhazzani et al. found that 63.6% and 43.7% of primary health center patients correctly identified thrombosis and hemorrhage, respectively, as types of strokes. The most reported risk factors were hypertension (55.8%), dyslipidemia (45.8%), and smoking (41.9%). Sudden severe headache (54.1%), dizziness (51.0%), and difficulty in speaking (44.3%) were the most frequently recognized symptoms [29]. Another study revealed that the mean knowledge of stroke risk among hypertensive patients was 10.73 ±3.53 while the mean knowledge of warning signs was 9.276±2.99 [30].

The main limitation was the smaller than desired sample size.

## Conclusions

The findings of the current study showed that less than half (43.1%) of the patients had a good awareness level regarding stroke and its related risk factors and warning signs. Older patients with high social levels (high education and income) and those with a family history of stroke had significantly higher awareness levels. As for factors associated with stroke, the most reported was high blood pressure, DM, cigarette smoking, and high cholesterol level. Regarding the awareness of stroke clinical presentations, 70.8% knew about speech disorders, 69.8% knew about weakness or disability to move one-half of the body, 64.4% know about decreased vision, and 62.9% knew about decreased sensation or inability to feel things. Finally, formal education was the sole predictor of good knowledge of the signs or symptoms of a stroke. So, further emphasis on the importance of formal education is required.
